# The association between depressive symptoms, physical activity, flow state, and resting-state EEG in college students

**DOI:** 10.3389/fpsyt.2026.1903553

**Published:** 2026-10-30

**Authors:** Tao Meng, Yan Rao, Zixian Wang, Feng Ding, Haiyan Wen, Sunwei Huang, Xing Wang, Peijin Li

**Affiliations:** 1 Shanghai Lixin University of Accounting and Finance, College of Physical Education and Health, Shanghai, China; 2 Shanghai Normal University Tianhua College, Shanghai, China; 3 Yangzhou University, School of Physical Education, Yangzhou, China; 4 Shanghai University of Sport, School of Physical Education, Shanghai, China; 5 Shanghai University of International Business and Economics, School of physical education and health, Shanghai, China; 6 Shanghai Educational Center of Science and Art, School Physical Education, Shanghai, China

**Keywords:** college students, depression symptoms, flow state, mediator model, physical activity, resting-state EEG

## Abstract

**Objective:**

Exploring the correlations among physical activity, flow state, and resting-state EEG in college students with depressive symptoms.

**Methods:**

A cross-sectional study was conducted among 216 college students. Participants completed questionnaires assessing depressive symptoms, physical exercise, and flow proneness, followed by a 5-min resting-state EEG recording. Group differences and associations were examined using independent-samples t-tests, χ² tests, Pearson correlations, and regression-based serial mediation analyses. The primary mediation models were adjusted for sex and self-rated physical health, with unadjusted models reported for comparison.

**Results:**

Physical exercise was associated with fewer depressive symptoms and greater flow proneness, and significant frontal EEG lateralization differences were observed between students with and without depressive symptoms. In the unadjusted mediation analyses, significant indirect effects were observed through Fp2/Fp1 δ- and α2-band lateralization and through the serial EEG lateralization–flow pathways. However, after adjustment for sex and self-rated physical health, the EEG-related indirect effects were no longer statistically significant in either the δ- or α2-band model. In contrast, the indirect effect through flow proneness alone remained significant in both adjusted models (Ind2), indicating that the flow-related indirect association was more robust to covariate adjustment than the EEG-related pathways.

**Conclusion:**

There is a close correlation among physical activities, EEG-specific indicators and the state of flow. However, whether the EEG-specific indicators play a mediating role remains to be further verified.

## 1 Introduction

Depressive symptoms among university students have emerged as a major public health concern, with impairments extending beyond core affective symptoms (such as depressed mood and anhedonia) to encompass broad cognitive domains, including attentional control deficits, working memory decline, and executive dysfunction (1). These cognitive impairments interact with affective symptoms, creating a vicious cycle that promotes symptom chronicity and social functional decline (2). Consequently, developing effective intervention strategies capable of simultaneously ameliorating both affective and cognitive symptoms is urgently needed. In recent years, the flow theory from positive psychology has provided a novel perspective. Flow, first proposed by Csikszentmihalyi, is defined as an optimal experiential state characterized by complete absorption and loss of self-consciousness when individuals engage in activities with balanced challenge-skill demands (3). Core features of this state include clear goals, immediate feedback, action-awareness merging, and complete concentration on the activity (3). From a neurophysiological perspective, flow represents a dynamic, task-dependent neural state involving fine-tuned regulation of prefrontal-parietal networks, particularly evident in motor performance requiring extensive practice and precise neurophysiological control (4, 5). The sustained attentional focus and immediate goal-directedness inherent to flow states directly target core cognitive-behavioral deficits in depression (4, 5). Notably, an individual’s flow proneness—the trait-level tendency to enter flow states—differs conceptually from the acute flow state experienced during specific activities (6). The former reflects stable individual difference characteristics potentially related to baseline brain functional features, whereas the latter represents a dynamic, context-dependent momentary experience (6). This conceptual distinction provides theoretical grounds for exploring the neural basis of flow from a resting-state brain function perspective. However, how depressive symptoms impair flow-generating capacity and the underlying neurophysiological mechanisms remain unclear, constituting the core scientific question this study aims to address.

Physical exercise provides an ideal behavioral model for examining these questions. Substantial high-quality evidence has established the causal therapeutic effect of physical exercise on depressive symptoms. Network meta-analyses demonstrate that exercise modalities including walking or jogging, yoga, and strength training exert significant antidepressant effects, particularly at higher intensities (7). Systematic reviews and meta-analyses further confirm that exercise should be considered an evidence-based treatment option for depression (8). Prospective meta-analyses have also revealed dose-response associations between physical activity and depression risk (9). However, the underlying psychological and neurophysiological factors mediating exercise’s antidepressant effects require further exploration. From a behavioral perspective, the activity characteristics of physical exercise (such as clear goal-setting, immediate performance feedback, and dynamic challenge-skill balance) may provide conducive contexts for flow experiences (6, 10). From a neurobiological perspective, exercise benefits derive from multilevel mechanisms: it promotes synthesis and release of neurotrophic factors (such as BDNF), optimizes neurotransmitter systems including dopaminergic and serotonergic pathways, and exerts anti-neuroinflammatory and neuroplasticity-promoting effects, particularly in brain regions critical for emotion and cognition such as the hippocampus and prefrontal cortex (11, 12). From an electrophysiological perspective, longitudinal intervention studies and experimental evidence indicate that regular physical exercise correlates with alterations in resting-state electroencephalographic (EEG) features: aerobic exercise interventions have shown enhanced prefrontal alpha power and leftward prefrontal alpha asymmetry (FAA), with these changes associated with symptom improvement in depressed individuals (13); cross-sectional comparisons reveal that long-term exercisers, compared to sedentary controls, exhibit stronger prefrontal-parietal theta synchronization associated with superior executive control (14). Although this evidence suggests physical exercise may influence EEG activity, the causal direction requires further verification. These observations indicate that physical exercise may create favorable neural conditions for flow experience generation by optimizing brain functional states. Therefore, exploring the associational patterns among physical exercise, brain activity, and flow represents a logical next step, contingent upon precise neuroimaging evidence.

Resting-state EEG provides unique advantages for noninvasively probing these associations at millisecond temporal resolution (15). Among oscillatory frequency bands, alpha rhythms (8–13 Hz, particularly the upper alpha/α2 subband 10–13 Hz) in the prefrontal-parietal system are considered highly relevant to flow-related cognitive-affective processes (5, 16). Specifically, task-state studies have found that flow states are accompanied by enhanced frontal midline theta, dynamic alpha modulation, and beta/gamma synchronization (4). Although flow is inherently a transient, performance-related emergent phenomenon, emerging research suggests that resting-state EEG features (such as baseline prefrontal FAA) may predict an individual’s flow proneness—the trait-level susceptibility to entering flow states (13, 14). This indicates that resting-state EEG may not directly reflect the flow state itself but rather the baseline functional organization pattern of the brain, which may influence an individual’s capacity to generate flow experiences in task contexts.

However, individuals with depressive symptoms exhibit characteristic resting-state EEG abnormalities: right prefrontal alpha activation dominance (negative FAA) reflects avoidance motivation predominance, while reduced prefrontal-parietal theta synchronization is associated with attentional control deficits (17). These abnormalities are present even in university students with subclinical depression (13, 14, 17). Nevertheless, it must be emphasized that the relationship between FAA and depression exhibits substantial between-study heterogeneity. Early meta-analyses, while finding associations between FAA and depression and anxiety, also highlighted the importance of variability and moderating effects (18). Subsequent reviews questioned the robustness of FAA as an independent diagnostic marker for major depressive disorder, emphasizing contradictory findings in the literature (19). The most recent meta-analyses further indicate that methodological heterogeneity, electrode selection, asymmetry index definition, and sample characteristics significantly influence FAA-depression associations (20). Nonetheless, theoretically, these EEG abnormalities may disrupt two core neural elements required for flow generation—left prefrontal-mediated approach motivation and prefrontal-parietal theta-supported sustained attentional control (5, 18, 21). Consequently, this study focuses on associations between resting-state EEG (particularly alpha lateralization) and flow proneness (rather than flow state), and how this association operates within the relationship between physical exercise and depressive symptoms.

Integrating the above evidence—physical exercise correlates with resting-state EEG changes, specific EEG patterns correlate with flow proneness, and EEG abnormalities in depression correlate with impaired flow capacity—this study aims to explore the associational patterns among physical exercise, resting-state EEG lateralization (focusing on alpha band, particularly the α2 subband), flow proneness, and depressive symptoms, and to examine statistical mediation relationships among these variables (18). It must be clarified that this study employs a cross-sectional design and cannot determine causal direction or temporal sequence among variables. All mediation analysis results should be interpreted as exploratory descriptions of potential associational pathways rather than estimates of genuine longitudinal mediation processes (22, 23). Although these bivariate relationships have been separately validated in different studies, their integrated examination within a single sample and systematic exploration of associational patterns remain unexplored—this constitutes the core innovation of this study.

This study adopts a cross-sectional survey design with university students experiencing depressive symptoms as participants, specifically examining: (1) Are there significant associations among physical exercise volume, resting-state EEG indices (primarily focusing on alpha lateralization, particularly the α2 subband 10–13 Hz; delta band 1–4 Hz as exploratory analysis), and flow proneness? (2) Do resting-state EEG lateralization indices and flow proneness show potential serial indirect associations in the relationship between physical exercise and depressive symptoms after accounting for sex and self-rated physical health? (3) If statistical mediation effects exist, which specific brain regions and frequency bands exhibit associated activity? This study will provide exploratory electrophysiological evidence for associational patterns among physical exercise, resting-state brain function, and flow proneness from an interdisciplinary perspective bridging positive psychology and cognitive neuroscience. These findings may provide a hypothesis-generating foundation for future longitudinal studies and intervention experiments, but the limitations of cross-sectional design necessitate cautious interpretation of all associations without causal inference. The value of this study lies not in demonstrating whether exercise is effective for depression (which is already well established) but in exploratorily identifying associational patterns of psychological traits and neurophysiological markers potentially related to this effect.

## 2 Research participants and methods

### 2.1 Participant recruitment

The sample size estimation for this study was based on the mediation effect model and was calculated using the G*Power software. The assumed effect size was moderate (f^2^ = 0.15), with a significance level of α = 0.05 and a statistical power of 0.80. Based on these settings, the estimated sample size was approximately 85 to 100 participants, which is suitable for a mediation model with a single path. This estimation is based on cross-sectional data and assumes a standard regression analysis model. This study was conducted based on voluntary participation through a mental health survey across seven universities in Shanghai’s Songjiang University Town, recruiting 236 college students via convenience sampling according to specified inclusion and exclusion criteria.

Inclusion criteria included: non-physical education majors; right-handed individuals; those in good physical health without clinically diagnosed psychiatric disorders; individuals with no history of psychotropic medication usage; no vigorous exercise within the 24 hours prior to testing; and no consumption of caffeinated or alcoholic beverages before the assessment.

Exclusion criteria consisted of: incomplete questionnaires, withdrawal from the testing process, and the presence of cardiovascular or organic diseases.

Participants were recruited from the general college student population. Depressive symptoms were assessed using the Beck Depression Inventory-II (BDI-II). Based on the BDI-II scores, participants with total scores >13 were classified into the depressive symptoms group, whereas those with total scores ≤13 were classified into the group without depressive symptoms. These classifications were used for subsequent group comparison analyses. This grouping reflects differences in self-reported depressive symptom severity and does not represent a clinical diagnosis of depressive disorders.

The study received ethical approval from the Ethics Committee of Shanghai University of Sport (Ethics Approval Number: 102772021RT004). We confirm that all methods were performed in accordance with the relevant guidelines and regulations by including a statement in the methods section to this effect. Before the experiment, all participants were made aware of the measurement procedures and potential risks, and they provided informed consent by signing the relevant forms. The participant recruitment process is illustrated in the **Supplementary Materials**.

This test was conducted from September to December 2023 at the Research Center for Sports and Health Promotion of Shanghai University of Sport, from 2 p.m. to 5 p.m. The test process is shown in the **Supplementary Materials**.

### 2.2 Research methods

#### 2.2.1 Questionnaire survey

Throughout the testing process, one principal investigator and two assistant researchers were responsible for data collection, operations, and supervision. All testing personnel were blind to the participants’ personal information. Participants were typically informed that the study aimed to evaluate the relationship between emotions and performance as reflected in EEG data, without divulging any specific hypotheses. Participants were made aware of the testing procedures and the precautions for EEG data collection. All data analysis was performed by a separate researcher who had no involvement in the intervention or data collection.

##### 2.2.1.1 Basic information questionnaire

The basic information collected from participants encompassed gender, age, height, weight, smoking and drinking habits, personal health status, interpersonal relationships, academic difficulty, and the frequency of social activities.

##### 2.2.1.2 Flow state scale

The Flow State Scale (FSS) was developed by Jackson and Marsh to investigate individuals’ flow states across various contexts. It has been revised and translated by Chinese scholars Hu Yongmei and Sun Yanlin (24). This study adopted the Chinese version translated by Hu Yongmei. The reliability and validity of this version were α = 0.792, and it is suitable for daily physical exercise. The total score is 180 points, consisting of 9 sub-dimensions (challenge-skill balance score, action-awareness integration score, clear goal score, precise feedback score, concentration, sense of control, loss of self-awareness, change in time perception, and experience of having a purpose).The flow state scale used in this study is specifically designed to measure the trait-like tendency of flow (the inclination toward a state of flow).

##### 2.2.1.3 Physical activity rating scale-3

The PARS-3 scale was created by Hashimoto Masuo, and for this survey, the revised Chinese version by Liang Deqing and others was utilized, demonstrating an internal consistency reliability ranging from 0.80 to 0.86, with a test-retest reliability of 0.82 (25). This scale primarily assesses the physical activity levels of non-physical education students over the past month. The evaluation is based on three dimensions: intensity, duration, and frequency. Intensity and frequency are scored on a scale of 1 to 5, while duration is scored from 0 to 4. Higher intensity, frequency, and duration correspond to higher scores, indicating an increased level of physical activity. The formula for calculating the level of physical activity is: intensity × duration × frequency, yielding a score range of 0 to 100. A score of ≤19 indicates low physical activity (insufficient exercise), 20–42 indicates moderate activity, and ≥nd indicates high activity. The Chinese version of the PARS demonstrates good validity for assessing physical activity levels in university students, with internal consistency coefficients ranging from 0.796 to 0.856 (26) and a test–retest reliability of 0.82 (27).

##### 2.2.1.4 Beck depression inventory-II

The Beck Depression Inventory-II (BDI-II) was developed by psychologist Aaron T. Beck and his colleagues. For this study, the revised Chinese version by Wang Zhen and others was utilized, demonstrating an internal consistency reliability of 0.94 and good test-retest reliability (28). This scale is designed to evaluate the severity of various depressive symptoms and consists of 21 items, each rated on a scale from 0 to 3. The overall score is the sum of the ratings for all 21 items, with a total score ranging from 0 to 13 indicating no depression, 14 to 19 indicating mild depression, 20 to 28 indicating moderate depression, and 29 to 63 indicating severe depression. The Cronbach’s α coefficient was 0.94, item–total correlations ranged from 0.56 to 0.82, and inter-item correlations ranged from 0.18 to 0.71. The test–retest reliability was 0.55, indicating satisfactory internal consistency of the scale (29, 30).

#### 2.2.2 EEG signal acquisition and preprocessing

EEG signals were recorded using an EEG device (NCERP-190012) from Shanghai Nuo Cheng Electric Co., Ltd. Electrodes were placed following the international 10/20 electrode placement system, utilizing 16 monopolar leads. The sampling frequency was set to 500 Hz, with high-pass filtering at 0.3 Hz, low-pass filtering at 30 Hz, and notch filtering at 50 Hz. The impedance between the electrodes and the skin was maintained below 5 kΩ. The selected electrode sites included Fp1 (left frontal), Fp2 (right frontal), F3 (left frontal mid), F4 (right frontal mid), C3 (left central), C4 (right central), P3 (left parietal), P4 (right parietal), O1 (left occipital), O2 (right occipital), F7 (left frontal side), F8 (right frontal side), T3 (left temporal mid), T4 (right temporal mid), T5 (left temporal back), and T6 (right temporal back). The ground electrode was placed at the mid-frontal (GND), and the reference electrodes were positioned on both mastoid processes (A1 and A2). The testing environment was a soundproof, well-ventilated, and suitably lit indoor setting, free from electromagnetic interference. Participants were seated, and testing personnel instructed them to close their eyes, remain relaxed, quiet, and alert, avoiding actions such as swallowing or teeth clenching. After achieving stability, EEG signals were recorded for 5 minutes.

Classic EEG analysis is usually performed within frequency bands below 30 Hz, which mainly include delta (1–4 Hz), theta (4–8 Hz), alpha1 (8-10.5 Hz), alpha2 (10.5–13 Hz), beta1 (13–20 Hz), and beta2 (20–30 Hz). A left-closed, right-open interval is used, with the critical value categorized in the high-frequency band. For examining differences between brain regions, the frontal region (Fp1-F3-F7, Fp2-F4-F8) is analyzed across these frequency bands.

Data preprocessing was carried out using EEGLAB in MATLAB 2023a. The specific steps for preprocessing were as follows: (1) Import continuous EEG data; (2) Localize electrodes; (3) Remove non-functional electrodes, Specifically, only the predefined 16 scalp EEG channels were retained for subsequent spectral/lateralization analyses; reference channels (A1/A2) and other non-target electrodes (e.g., midline/auxiliary channels) were excluded from statistical analyses. For retained channels, “non-functional” was defined as abnormally high impedance, prolonged flatline signals, persistent saturation/clipping, or excessive noise (e.g., unusually high variance and/or atypical spectra).; (4) Reduce the sampling rate to 256 Hz; (5) Apply high-pass filtering at 0.1 Hz and low-pass filtering at 30 Hz; (6) Use an average reference electrode; (7) Inspect the waveform for bad electrodes, and interpolate if necessary; (8) Segment the continuous EEG data into 2-second epochs; (9)Apply Independent Component Analysis (ICA) in EEGLAB (MATLAB) using the runica (Infomax) algorithm to identify and remove non-neural artifacts, including eye movements, blinks, electromyographic activity, electrocardiographic activity, line noise, and other non-physiological signals; (10) Artifact-related components were identified by visual inspection of their scalp topographies, time courses, and power spectra, and the corresponding components were removed prior to EEG signal reconstruction; (11) Reject epochs containing EEG amplitudes exceeding ±100 μV; (12) Manually reject EEG segments showing obvious signal drift or residual artifacts; (13) Save the preprocessed EEG data. An average of approximately 135 clean epochs (approximately 4.5 min of EEG data) was retained per participant for subsequent analyses.

To quantify hemispheric lateralization, we calculated the lateralization index (LI) (31, 32) as


$$LI=\frac{R-L}{R+L}$$

The index ranges from [–1, 1], with positive values indicating right-side dominance (right lateralization) and negative values reflecting left-side dominance (left lateralization). The absolute value of the index represents the degree of lateralization. In this study, lateralization indices (LI) for Fp2/Fp1, F4/F3, and F8/F7 were calculated across δ, θ, α1, α2, β1, and β2 frequency bands. Given the inverse relationship between α power and cortical activation, an increase in α band LI was interpreted as reflecting relatively enhanced activation of the left cortical region.

The selection of EEG lateralization indices for subsequent analyses was exploratory. Specifically, lateralization indices were examined for three bilateral frontal electrode pairs (Fp2/Fp1, F4/F3, and F8/F7) across six frequency bands (δ, θ, α1, α2, β1, and β2), resulting in 18 EEG comparisons. To account for multiple testing, a Bonferroni-corrected significance threshold was applied to these 18 comparisons (α = 0.05/18 = 0.0028). EEG indices showing group differences that remained significant after correction were considered exploratory candidate markers for subsequent analyses rather than pre-specified primary EEG endpoints.

### 2.3 Statistical analysis

Data analysis was performed using SPSS 29.0. Measurement data following or approximating a normal distribution were reported as mean ± standard deviation (M ± SD), while categorical data were presented as frequencies. Differences in categorical variables were assessed using the χ2 test, and group comparisons were conducted via independent sample t-tests. Pearson correlation analysis was performed to examine the associations between physical exercise, depressive symptoms, flow state, and frontal lobe lateralization. For the exploratory comparisons of EEG lateralization indices, 18 comparisons were conducted across three bilateral frontal electrode pairs (Fp2/Fp1, F4/F3, and F8/F7) and six frequency bands (δ, θ, α1, α2, β1, and β2). A Bonferroni correction was applied to control the family-wise Type I error rate, yielding a corrected significance threshold of α = 0.05/18 = 0.0028. EEG lateralization indices showing significant group differences after Bonferroni correction were subsequently examined in the correlation and serial mediation analyses. Separate serial mediation models were tested for each candidate EEG index. Regression-based serial mediation analyses were conducted using the PROCESS macro (Model 6) in SPSS 29.0. In the mediation models, physical exercise level measured by PARS-3 was specified as the independent variable (X), frontal lobe lateralization index was specified as the first mediator (M1), flow state was specified as the second mediator (M2), and depressive symptoms assessed by BDI-II were specified as the outcome variable (Y). The model estimated the direct effect, total effect, and specific indirect effects through the proposed mediation pathways using ordinary least squares regression. Indirect effects were evaluated using 5,000 bootstrap resamples with bias-corrected 95% confidence intervals (CIs). An indirect effect was considered statistically significant when the corresponding confidence interval did not include zero. Given the significant group differences in sex and self-rated physical health, the primary mediation models were estimated with sex and self-rated physical health included as covariates. The corresponding unadjusted models were retained as exploratory comparison analyses. To evaluate sample size adequacy, a G*Power sensitivity analysis was conducted for linear multiple regression (fixed model, R² deviation from zero). With n = 216, α = 0.05, 3 predictors, and power = 0.80, the minimum detectable effect size was f² = 0.051 (R² ≈ 0.049). In addition, a *post-hoc* Monte Carlo power analysis was conducted for the serial indirect effect (Xfectctde, using the observed path coefficients and their standard errors, with 5,000 simulations and a two-tailed α = 0.05. All statistical tests were two-tailed. Unless otherwise specified, statistical significance was evaluated at α = 0.05; for the 18 exploratory EEG lateralization comparisons, the Bonferroni-corrected threshold was α = 0.0028.

The EEG marker screening and subsequent analyses involving the selected EEG indices were considered exploratory because candidate EEG markers were identified and further evaluated within the same sample. Accordingly, these analyses were interpreted as hypothesis-generating rather than independent confirmatory evidence.

## 3 Results

### 3.1 Basic information

To explore the potential factors influencing depressive symptoms and their associations with the basic characteristics of college students, participants were categorized into two groups: participants were classified into a group with depressive symptoms (n = 127) and a group without depressive symptoms (n = 89) according to their BDI-II scores. Significant group differences were observed for gender and self-rated physical health. (P<0.05). These findings indicate that gender and self-rated physical health were associated with depressive symptom levels among college students. (See **Table 1**).

**Table 1:** Group comparisons of participant characteristics between the depressive symptoms and group without depressive symptoms.

Variable	All (n=216)	Group with depressive symptoms(n=127)	Group without depressive symptoms (n = 89)	Difference examination
Age/Years	19.12 ± 1.04	19.11 ± 1.06	19.13 ± 1.01	t=-0.171, p=0.865
BMI/(Kg/m^2^)	21.48 ± 3.93	21.61 ± 4.35	21.29 ± 3.24	t=0.608, p=0.544
Gender				*χ*^2^ = 28.400, p<0.001
Man	99	39	60	
Women	117	88	29	
Alcohol drinking habit				*χ*^2^ = 1.105, p=0.293
Yes	36	24	12	
No	180	103	77	
Smoking habit				*χ*^2^ = 2.856, p=0.091
Yes	4	4	0	
No	212	123	89	
Frequency of social activities				*χ*^2^ = 1.488, p=0.685
Everyday	2	1	1	
4 to 6 times per week	6	4	2	
1–3 times per week	151	85	66	
Never	57	37	20	
Self-assessment of physical condition				*χ*^2^ = 14.317, p=0.006
Very good	26	11	15	
good	82	40	42	
Normal	83	56	27	
Bad	21	16	5	
Very bad	4	4	0	
Interpersonal relationship				*χ*^2^ = 7.979, p=0.092
Very good	38	18	20	
good	93	50	43	
Normal	79	56	23	
Bad	4	2	2	
Very bad	2	1	1	
Learning difficulty				*χ*^2^ = 7.600, p=0.107
Very difficult	5	5	0	
Difficult	30	15	15	
Normal	128	76	52	
Easy	42	22	20	
Very easy	11	9	2	

### 3.2 Multiple linear regression analysis of risk factors for depressive symptoms

To investigate the factors influencing depressive symptoms among college students, the total BDI-II score was entered as the dependent variable, and a stepwise multiple linear regression analysis was performed with gender, self-rated physical health, flow state, and physical exercise as candidate predictors (**Table 2**). The final model explained 40.6% of the variance in depressive symptoms (R² = 0.406, adjusted R² = 0.397, F(3,212) = 48.281, p < 0.001). Flow state was retained as the strongest predictor and was negatively associated with depressive symptoms (B = -0.263, SE = 0.030, β = -0.504, t = -8.730, p < 0.001). Physical exercise also showed a significant negative association with depressive symptoms (B = -0.115, SE = 0.052, β = -0.126, t = -2.196, p = 0.029). Gender was identified as a significant positive predictor (B = 3.508, SE = 1.117, β = 0.176, t = 3.140, p = 0.002), indicating that female participants had, on average, BDI-II scores approximately 3.51 points higher than male participants after accounting for the other predictors in the model. Collinearity diagnostics indicated no evidence of multicollinearity (all VIF ≤ 1.19).

**Table 2:** Multiple linear regression predicting depressive symptoms (BDI-II): model fit and regression coefficients of the final model.

Variable	R²	Adjusted R²	F	p	B	SE	Beta	t	p	95%CI	Collinearity statistics	
											Tolerance	VIF
Model	0.406	0.397	48.281	<0.001								
FSS					-0.263	0.030	-0.504	-8.730	<0.001	[-0.322,-0.204]	0.841	1.189
Gender					3.508	1.117	0.176	3.140	0.002	[1.305,5.710]	0.892	1.121
PARS-3					-0.115	0.052	-0.126	-2.196	0.029	[-0.219,-0.012]	0.850	1.176

The dependent variable was the total score of BDI-II; the order of entry of independent variables was flow experience → gender (0 = male, 1 = female) → physical exercise.

### 3.3 Differences in frontal lateralization among groups

This section aimed to identify frontal EEG lateralization indices that differentiated students with versus without depressive symptoms. These group-differentiating indices were treated as exploratory candidate markers and further evaluated in subsequent analyses. As shown in **Table 3**, the depressive-symptom group had significantly lower flow-state scores than the no-depressive-symptom group (t = −9.650, p < 0.001). Physical exercise scores were also significantly lower in the depressive-symptom group (t = −5.069, p < 0.001). For the frontal EEG lateralization indices, 18 group comparisons were conducted across three bilateral electrode pairs (Fp2/Fp1, F4/F3, and F8/F7) and six frequency bands (δ, θ, α1, α2, β1, and β2). In the frontopolar Fp2/Fp1 pair, the depressive-symptom group showed significantly lower lateralization indices in the δ, θ, α1, and α2 bands than the no-depressive-symptom group (all unadjusted p < 0.001). After Bonferroni correction for the 18 EEG comparisons (corrected significance threshold: α = 0.05/18 = 0.0028), all four Fp2/Fp1 differences remained statistically significant. No significant between-group differences were observed for the Fp2/Fp1 β1 or β2 bands, or for any of the six frequency bands at F4/F3 or F8/F7 (all p > 0.05).

**Table 3:** Differences in lateralization of the frontal regions among different groups.

Variable	Mean ± SD		T	P
	Depression symptom group(n=127)	Non-depressed symptom group (n = 89)		
FSS	98.71 ± 14.79	120.39 ± 17.21	-9.650	<0.001
PARS-3	6.79 ± 7.64	14.60 ± 13.05	-5.069	<0.001
Fp2/Fp1-δ	-0.29 ± 0.19	-0.12 ± 0.17	-6.764	<0.001
Fp2/Fp1-θ	-0.30 ± 0.23	-0.11 ± 0.13	-7.755	<0.001
Fp2/Fp1-α1	-0.35 ± 0.31	-0.09 ± 0.14	-8.426	<0.001
Fp2/Fp1-α2	-0.35 ± 0.31	-0.05 ± 0.17	-9.023	<0.001
Fp2/Fp1-β1	-0.21 ± 0.28	-0.24 ± 0.25	0.859	0.392
Fp2/Fp1-β2	-0.19 ± 0.28	-0.22 ± 0.25	0.920	0.358
F4/F3-δ	0.00 ± 0.15	-0.019 ± 0.15	1.053	0.294
F4/F3-θ	0.09 ± 0.24	0.05 ± 0.21	1.114	0.266
F4/F3-α1	0.16 ± 0.37	0.12 ± 0.30	0.899	0.370
F4/F3-α2	0.18 ± 0.35	0.14 ± 0.31	0.743	0.458
F4/F3-β1	0.15 ± 0.33	0.11 ± 0.30	0.817	0.415
F4/F3-β2	0.17 ± 0.33	0.13 ± 0.32	1.069	0.286
F8/F7-δ	-0.01 ± 0.14	-0.03 ± 0.15	0.928	0.354
F8/F7-θ	-0.00 ± 0.13	-0.02 ± 0.16	1.195	0.233
F8/F7-α1	0.00 ± 0.17	-0.03 ± 0.20	1.248	0.213
F8/F7-α2	0.03 ± 0.16	-0.01 ± 0.23	1.193	0.235
F8/F7-β1	0.02 ± 0.15	-0.01 ± 0.20	1.351	0.179
F8/F7-β2	0.04 ± 0.17	-0.01 ± 0.23	1.644	0.102

Eighteen EEG lateralization comparisons were evaluated (3 electrode pairs × 6 frequency bands). A Bonferroni-corrected significance threshold of α = 0.0028 (0.05/18) was applied. Reported p values are unadjusted.

According to the definition LI = (R−L)/(R+L), lower or more negative LI values indicate a relative shift toward left-side power dominance. For the α bands specifically, because α power is inversely related to cortical activation, lower α-band LI values may correspond to relatively greater right-sided cortical activation. These findings were considered exploratory and were used to identify candidate EEG indices for subsequent analyses.

### 3.4 Correlation analysis of depression symptoms, flow state, physical exercise, and frontal lateralization across frequency bands

As shown in **Figure 1**, the correlation heatmap highlights a coherent pattern linking behavioral measures (flow state and physical exercise) with depressive symptoms, and suggests that frontal polar lateralization (Fp2/Fp1) is meaningfully associated with these variables. Overall, higher flow state and physical exercise are associated with lower depression symptoms, while the frontal lateralization indices show selective associations consistent with the proposed pathway. Flow state (r = -0.597, p<0.001) and physical exercise (r = -0.347, p<0.001) are both significantly negatively correlated with depression symptoms. Moreover, the lateralization indices for the δ band (r = -0.354, p<0.001) and the α2 band (r = -0.428, p<0.01) within the frontal region Fp2/Fp1 also exhibit significant negative correlations with depression symptoms. In contrast, the lateralization indices for the δ band (r = 0.233, p<0.01; r = 0.176, p<0.05) and the α2 band (r = 0.330, p<0.05; r = 0.143, p<0.05) in Fp2/Fp1 reveal significant positive correlations with both flow state and physical exercise. These findings suggest a consistent pattern in Fp2/Fp1 such that higher flow state and physical exercise are associated with more leftward frontal polar lateralization, whereas greater depressive symptom severity is associated with more rightward lateralization, particularly in the δ and α2 bands (see **Figure 1**).

**Figure 1:**
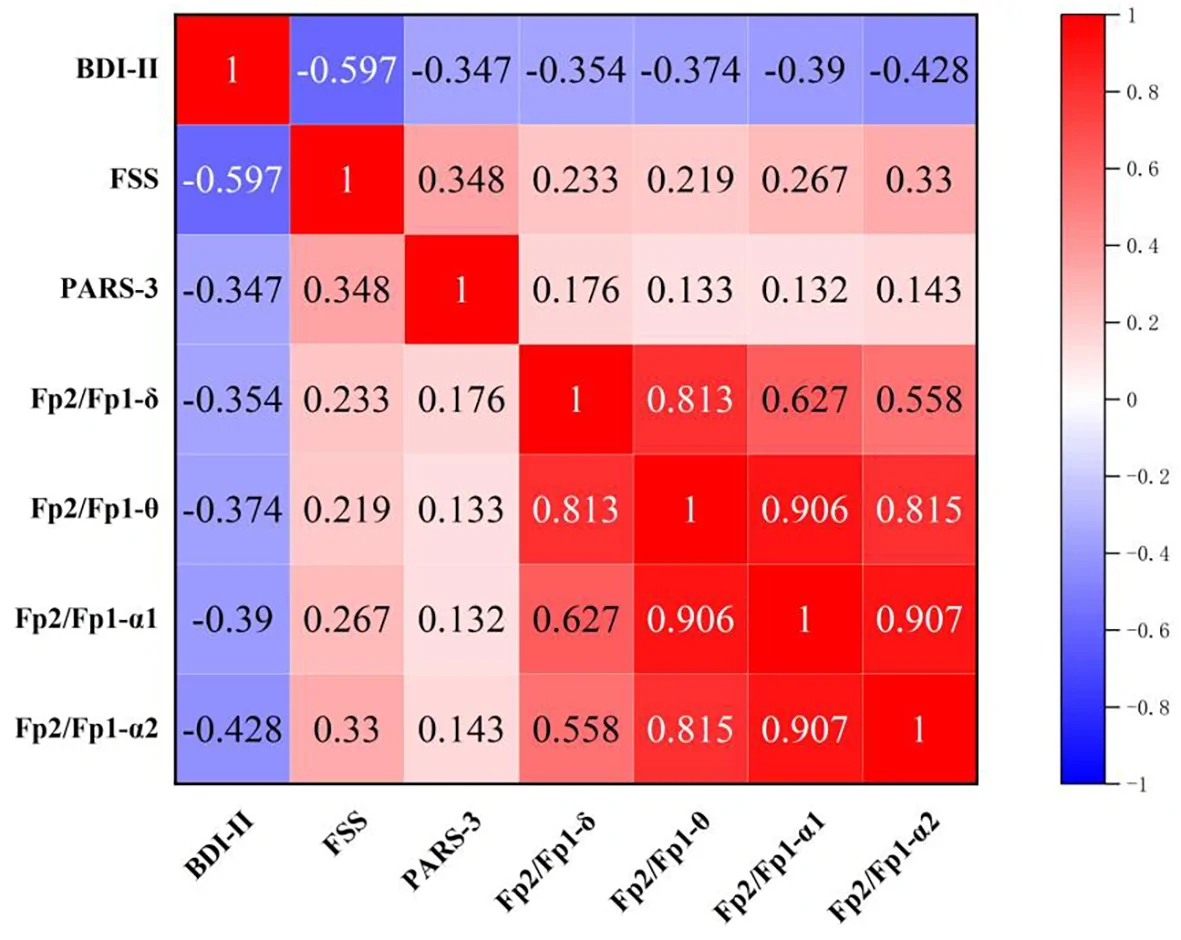
Correlation heatmap of depressive symptoms, flow state, physical exercise and lateralization of each frequency band in the frontal region. Correlation analyses were exploratory, and the reported p values are unadjusted; these results were therefore interpreted as descriptive, hypothesis-generating associations.

### 3.5 Indirect-effect analyses of physical exercise, frontal EEG lateralization, flow proneness, and depressive symptoms

Separate serial indirect-effect models were examined for the four Fp2/Fp1 lateralization indices (δ, θ, α1, and α2) that remained significant after Bonferroni correction in the group comparisons. Given the significant group differences in sex and self-rated physical health, models adjusted for these two variables were treated as the primary analyses. In the covariate-adjusted models, the EEG-related serial indirect effects were not statistically significant. The corresponding unadjusted analyses are reported below for comparison and should be interpreted as exploratory findings.

#### 3.5.1 Chain mediation model via frontal δ-band lateralization (Fp2/Fp1) and flow state

In the primary model adjusted for sex and self-rated physical health, the serial indirect effect of physical exercise on depressive symptoms through Fp2/Fp1 δ-band lateralization and flow proneness was not statistically significant (Effect = −0.0044, BootSE = 0.0046, 95% bootstrap CI [−0.0152, 0.0030]). The specific indirect effect through δ-band lateralization alone was also not statistically significant (Effect = −0.0144, 95% bootstrap CI [−0.0430, 0.0107]), whereas the indirect effect through flow proneness alone remained significant (Effect = −0.1007, 95% bootstrap CI [−0.1594, −0.0506]). Under the covariate-adjusted regression-based mediation framework, the model explained 11.9% of the variance in Fp2/Fp1 δ-band lateralization (M1; R² = 0.119), 21.0% of the variance in flow proneness (M2; R² = 0.210), and 44.6% of the variance in depressive symptoms (Y; R² = 0.446). Thus, although the indirect association through flow proneness remained statistically supported, the EEG-related indirect effects observed in the unadjusted δ-band model were not maintained after adjustment for sex and self-rated physical health (**Figures 2A, B**).

**Figure 2:**
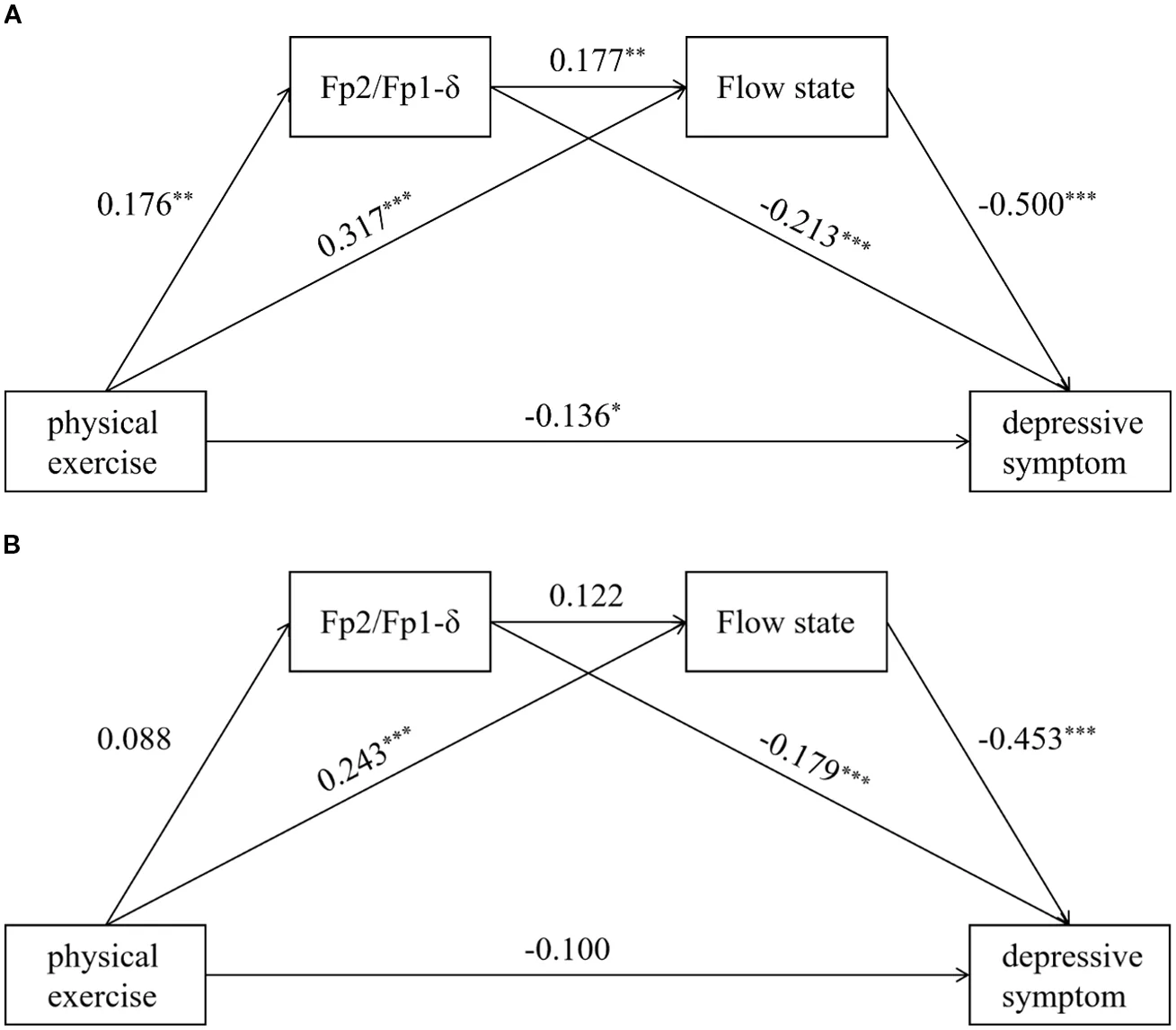
**(A)** Unadjusted serial indirect-effect model involving physical exercise, Fp2/Fp1 δ-band lateralization, flow proneness, and depressive symptoms. **(B)** Covariate-adjusted serial indirect-effect model involving physical exercise, Fp2/Fp1 δ-band lateralization, flow proneness, and depressive symptoms. The coefficients presented on each pathway represent standardized regression coefficients (β) from the PROCESS Model 6 serial mediation analysis. "*", "**" and "***" respectively indicate p < 0.05, p < 0.01 and p < 0.001.

For comparison, the unadjusted model yielded significant specific indirect effects through δ-band lateralization alone, flow proneness alone, and the serial δ-band lateralization–flow pathway (**Tables 4**, **5A**, **5B**). However, given the loss of the EEG-related indirect effects after covariate adjustment, together with the susceptibility of frontopolar δ activity to residual ocular and slow-drift artifacts, the unadjusted δ-band findings should be regarded as exploratory and hypothesis-generating.

**Table 4:** Total and direct effects of physical exercise on depressive symptoms in the unadjusted and covariate-adjusted models involving frontal pole δ-band lateralization and flow state.

Model	Effect size	Effect	SE	t	p	LLCI	ULCI	Completely standardized effects
Unadjusted	Total effect	-0.3171	0.0586	-5.4148	<0.001	-0.4325	-0.2017	-0.3471
	Direct effect	-0.1241	0.0512	-2.4238	0.0162	-0.2250	-0.0232	-0.1358
Covariate- adjusted	Total effect	-0.2106	0.0580	-3.6326	0.0004	-0.3249	-0.0963	-0.2305
	Direct effect	-0.0911	0.0514	-1.7728	0.0777	-0.1925	0.0102	-0.0998

The covariate-adjusted model included sex and self-rated physical health as covariates; the unadjusted model included no covariates.

**Table 5A:** Specific indirect effects of physical exercise on depressive symptoms via frontal pole δ-band lateralization and flow state: unadjusted model.

Path	Effect	Boot SE	Boot LLCI	Boot ULCI	Completely standardized effects
TOTAL	-0.1930	0.0358	-0.2687	-0.1274	-0.2113
Ind1: Physical exercise → Fp2/Fp1-δ → Depression symptoms	-0.0342	0.0161	-0.0697	-0.0065	-0.0375
Ind2: Physical exercise → Flow state → Depression symptoms	-0.1446	0.0292	-0.2083	-0.0926	-0.1582
Ind3: Physical exercise → Fp2/Fp1-δ → Flow state → Depression symptoms	-0.0142	0.0070	-0.0297	-0.0023	-0.0156

No covariates were included in the model.

**Table 5B:** Specific indirect effects of physical exercise on depressive symptoms via frontal pole δ-band lateralization and flow state: covariate-adjusted model.

Path	Effect	Boot SE	Boot LLCI	Boot ULCI	Completely standardized effects
TOTAL	-0.1195	0.0348	-0.1909	-0.0540	-0.1308
Ind1: Physical exercise → Fp2/Fp1-δ → Depression symptoms	-0.0144	0.0135	-0.0430	0.0107	-0.0157
Ind2: Physical exercise → Flow state → Depression symptoms	-0.1007	0.0277	-0.1594	-0.0506	-0.1102
Ind3: Physical exercise → Fp2/Fp1-δ → Flow state → Depression symptoms	-0.0044	0.0046	-0.0152	0.0030	-0.0049

The model was adjusted for sex and self-rated physical health.

#### 3.5.2 Chain mediation model via frontal α2-band lateralization (Fp2/Fp1) and flow state

In the primary model adjusted for sex and self-rated physical health, the serial indirect effect of physical exercise on depressive symptoms through Fp2/Fp1 α2-band lateralization and flow proneness was not statistically significant, as the 95% bootstrap confidence interval included zero. The specific indirect effect through α2-band lateralization alone was also not statistically significant, whereas the indirect effect through flow proneness alone remained significant. Consistent with the absence of a significant EEG-related serial indirect effect, physical exercise was not significantly associated with Fp2/Fp1 α2-band lateralization after covariate adjustment (B = 0.0010, SE = 0.0019, p = 0.588). In contrast, Fp2/Fp1 α2-band lateralization remained significantly associated with flow proneness (B = 14.3322, SE = 4.0753, p = 0.001) and depressive symptoms (B = −7.1590, SE = 1.8557, p < 0.001).

Under the covariate-adjusted regression-based mediation framework, the model explained 12.3% of the variance in Fp2/Fp1 α2-band lateralization (M1; R² = 0.123), 24.2% of the variance in flow proneness (M2; R² = 0.242), and 45.7% of the variance in depressive symptoms (Y; R² = 0.457). Thus, although α2-band lateralization remained associated with flow proneness and depressive symptoms, the EEG-related indirect effects observed in the unadjusted model were not maintained after adjustment for sex and self-rated physical health (**Figures 3A, B**).

**Figure 3:**
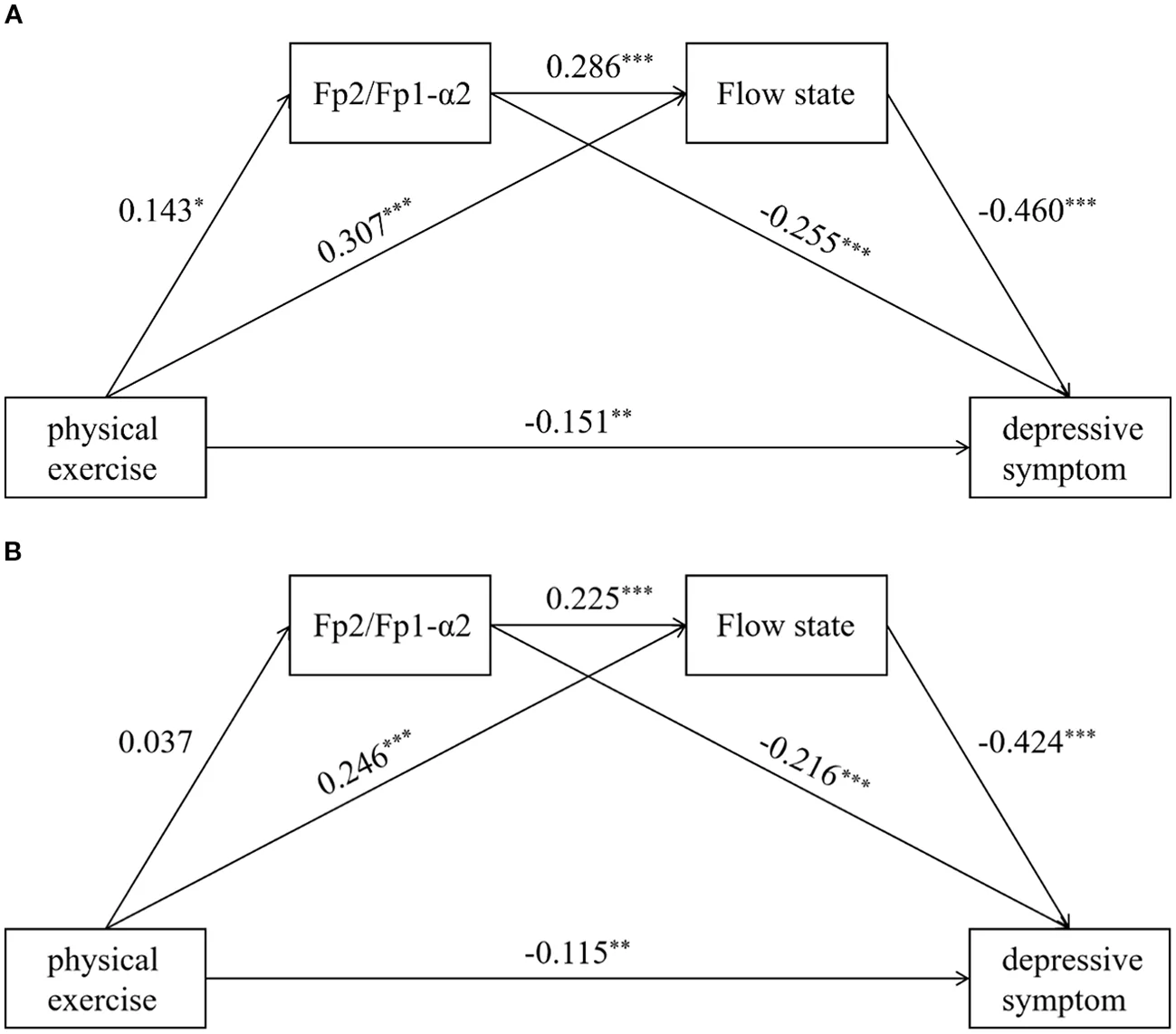
**(A)** Unadjusted serial indirect-effect model involving physical exercise, Fp2/Fp1 α2-band lateralization, flow proneness, and depressive symptoms. **(B)** Covariate-adjusted serial indirect-effect model involving physical exercise, Fp2/Fp1 α2-band lateralization, flow proneness, and depressive symptoms. The coefficients presented on each pathway represent standardized regression coefficients (β) from the PROCESS Model 6 serial mediation analysis. "*", "**" and "***" respectively indicate p < 0.05, p < 0.01 and p < 0.001.

For comparison, the unadjusted model yielded significant indirect effects through α2-band lateralization alone, flow proneness alone, and the serial α2-band lateralization–flow pathway (**Tables 6**, **7A**, **7B**). Given that the EEG-related indirect effects were not maintained after covariate adjustment, these unadjusted findings should be interpreted as exploratory rather than as evidence of a robust electrophysiological mediation pathway.

**Table 6:** Total and direct effects of physical exercise on depressive symptoms in the unadjusted and covariate-adjusted models involving frontal pole α2-band lateralization and flow state.

Model	Effect size	Effect	SE	t	p	LLCI	ULCI	Completely standardized effects
Unadjusted	Total effect	-0.3171	0.0586	-5.4148	<0.001	-0.4325	-0.2017	-0.3471
	Direct effect	-0.1375	0.0503	-2.7338	0.0068	-0.2366	-0.0383	-0.1505
Covariate- adjusted	Total effect	-0.2106	0.0580	-3.6326	0.0004	-0.3249	-0.0963	-0.2305
	Direct effect	-0.1050	0.0508	-2.0649	0.0402	-0.2052	-0.0048	-0.1149

The covariate-adjusted model included sex and self-rated physical health as covariates; the unadjusted model included no covariates.

**Table 7A:** Specific indirect effects of physical exercise on depressive symptoms via frontal pole α2-band lateralization and flow state: unadjusted model.

Path	Effect	Boot SE	Boot LLCI	Boot ULCI	Completely standardized effects
TOTAL	-0.1797	0.0365	-0.2574	-0.1120	-0.1967
Ind1: Physical exercise → Fp2/Fp1-α2 → Depression symptoms	-0.0334	0.0177	-0.0722	-0.0011	-0.0375
Ind2: Physical exercise → Flow state → Depression symptoms	-0.1290	0.0263	-0.1846	-0.0817	-0.1412
Ind3: Physical exercise → Fp2/Fp1-α2 → Flow state → Depression symptoms	-0.0172	0.0087	-0.0358	-0.0006	-0.0183

No covariates were included in the model.

**Table 7B:** Specific indirect effects of physical exercise on depressive symptoms via frontal pole α2-band lateralization and flow state: covariate-adjusted model.

Path	Effect	Boot SE	Boot LLCI	Boot ULCI	Completely standardized effects
TOTAL	-0.1056	0.0372	-0.1843	-0.0366	-0.1156
Ind1: Physical exercise → Fp2/Fp1-α2 → Depression symptoms	-0.0073	0.0164	-0.0431	0.0231	-0.0080
Ind2: Physical exercise → Flow state → Depression symptoms	-0.0951	0.0256	-0.1516	-0.0509	-0.1041
Ind3: Physical exercise → Fp2/Fp1-α2 → Flow state → Depression symptoms	-0.0032	0.0070	-0.0177	0.0106	-0.0035

The model was adjusted for sex and self-rated physical health.

In covariate-adjusted sensitivity analyses including sex and self-rated physical health, the bootstrap confidence intervals for the EEG-related serial indirect effects included zero, whereas the indirect effect through flow remained statistically supported.

## 4 Discussion

The present findings reveal significant correlations among depressive symptoms, flow proneness, physical exercise, and frontopolar (Fp1/Fp2) EEG lateralization. Specifically, flow proneness and physical exercise were negatively correlated with depressive symptoms, whereas Fp2/Fp1 α2 lateralization was negatively correlated with depressive symptoms and positively correlated with both flow proneness and physical exercise. These associational patterns indicate that more severe depressive symptoms are associated with more rightward frontopolar lateralization, whereas higher flow proneness and physical exercise levels are associated with more leftward lateralization. It is crucial to clarify that this study employed the Flow State Scale (FSS) to measure flow, which actually assesses flow proneness—the trait-level tendency to enter flow states—rather than acute flow states experienced during actual physical activity (6). This conceptual distinction is critical as it determines the interpretive scope of our findings. Our results indicate that higher self-reported physical exercise levels correlate with higher flow proneness traits, which may reflect: (1) individuals who regularly engage in exercise develop higher flow proneness through accumulated positive experiences; (2) individuals with high flow proneness are more willing to engage in and persist with exercise; or (3) both are influenced by common third variables (such as personality traits or intrinsic motivation). Only through real-time measurement of flow states in actual exercise contexts, combined with longitudinal or intervention designs, can the exercise-flow relationship be more precisely understood.

However, it must be particularly noted that all significant EEG findings in this study originated from frontopolar electrodes (Fp1/Fp2). These electrode locations are highly susceptible to contamination from eye movements (including macro- and microsaccades), blinks, and frontal electromyographic activity (33, 34). Although we employed independent component analysis (ICA) for artifact removal, the low-density EEG system (only 16 channels) limited spatial localization capacity, precluding complete exclusion of residual artifact influences (33, 34). Furthermore, the 16-channel EEG system severely constrains precise source localization and network interpretation capabilities; therefore, our discussion of “prefrontal-related activity” should be understood as observational descriptions based on scalp electrodes rather than precise localization of cortical source activity (33). These measurement limitations require cautious interpretation of the lateralization patterns observed at Fp1/Fp2, which may partially reflect eye movement pattern differences associated with emotional states rather than purely cortical neural activity (34).

It must be emphasized that the observed associations between frontopolar α2 lateralization and physical exercise, flow proneness, and depressive symptoms should be understood as exploratory findings rather than validation of an established biomarker (16). The literature on the relationship between prefrontal alpha asymmetry and depression exhibits substantial heterogeneity and contradictory findings (19, 20). Methodological factors (such as electrode position, reference scheme, frequency band definition, sample characteristics) may influence both the strength and direction of FAA-depression associations. The frontopolar (Fp1/Fp2) position and α2 frequency band (10–13 Hz) definition used in this study represent specific choices, and results cannot be directly generalized to other electrode positions or frequency definitions (16). Therefore, α2 lateralization index (α2-LI) in this study should be regarded as an exploratory electrophysiological candidate marker potentially compatible with theoretical frameworks of approach motivation and emotion regulation, but currently insufficient as a basis for clinical diagnosis or mechanistic inference, requiring replication in independent samples.

It should be noted that we also observed associations between frontopolar delta lateralization and physical exercise, flow proneness, and depressive symptoms. However, this finding should be regarded as highly exploratory and hypothesis-generating. The delta band in wakeful resting states is typically associated with sleep, deep relaxation, or certain pathological states, and its association with flow proneness and depressive symptoms during wakeful rest lacks adequate theoretical explanatory frameworks. Moreover, low-frequency delta signals are highly susceptible to contamination from eye movements (particularly slow eye movements) and electromyographic artifacts, a problem exacerbated by the frontopolar electrode position. Although ICA artifact removal was performed, the limitations of our 16-channel low-density system preclude complete confidence that these delta signals are free from residual artifacts. Therefore, the frontopolar delta lateralization finding requires independent verification under the following conditions: (1) use of high-density EEG systems (at least 64 channels); (2) more rigorous artifact control and visual inspection; (3) replication in multiple independent samples; and (4) provision of theoretical explanatory frameworks. Until adequately verified, we recommend readers regard the delta lateralization finding as preliminary observation requiring further exploration rather than a reliable marker.

Our mediation analyses revealed three statistical mediation pathways (primarily based on α2 lateralization) in the negative association between physical exercise and depressive symptoms: (1) physical exercise → α2 lateralization → depressive symptoms; (2) physical exercise → flow proneness → depressive symptoms; and (3) physical exercise → α2 lateralization → flow proneness → depressive symptoms. These results suggest that the association between physical exercise and depressive symptoms is not entirely direct but partially transmitted through frontopolar α2 lateralization and flow proneness, with a serial associational pattern of “α2 lateralization → flow proneness” (35–37) However, because the EEG-related serial indirect effect was attenuated in the covariate-adjusted sensitivity analysis, this pattern should be considered exploratory rather than evidence of an established mediating pathway. It must be emphasized that these mediation analysis results derive from cross-sectional data and should be interpreted as exploratory descriptions of potential associational pathways rather than estimates of genuine longitudinal mediation processes (22, 23). Cross-sectional mediation models may provide biased estimates of longitudinal mediation processes; even when statistically significant, they cannot be interpreted as true mediation processes (38).

This study found that α2 lateralization at frontopolar sites (Fp1/Fp2) correlates with physical exercise, flow proneness, and depressive symptoms. However, it must be emphasized that recent studies in similar populations have reported different EEG associational patterns, highlighting the complexity and heterogeneity of this field (21). Zhao et al. examined the relationship between resting-state EEG and physical exercise in university students with depressive symptoms, finding associations primarily involving alpha1 lateralization at dorsolateral prefrontal sites (F3/F4) and theta/alpha1 activity at lateral prefrontal sites (F7/F8), as well as differences in central, parietal, and posterior temporal regions (14). That study’s EEG associational pattern did not particularly emphasize frontopolar regions or the α2 band, exhibiting significant divergence from the present findings (35). Similarly, Liu et al. (2024), using resting-state EEG from parietal regions, examined immediate effects of single-session aerobic exercise at different intensities on depressive symptoms in university students, reporting effects of exercise on parietal α2 and β1 lateralization (39). That study focused on parietal rather than prefrontal regions and addressed acute exercise effects rather than habitual exercise levels. Importantly, Liu et al. also noted that exercise intensity effects on depressive symptoms and EEG lateralization remain inconsistent in the literature.

The present study is positioned as an exploratory associational investigation rather than an intervention mechanism study. Substantial causal evidence already demonstrates that physical exercise can effectively improve depressive symptoms (7–9). Building upon this, our study attempts to exploratorily identify associational patterns of psychological and neurophysiological factors potentially related to this effect. These cross-sectional associations may provide hypotheses for future longitudinal research: for example, does exercise intervention alter EEG patterns, flow proneness, and depressive symptoms in a specific temporal sequence? Only through multiwave longitudinal measurement or randomized controlled intervention designs can these causal and temporal questions be answered (40).

In summary, within a cross-sectional design framework, this study explored associational patterns among physical exercise, frontopolar EEG lateralization (primarily α2 band), flow proneness, and depressive symptoms. Results revealed statistically significant direct and indirect (mediated) associational patterns among these variables, suggesting that the negative association between physical exercise and depressive symptoms may be partially transmitted through resting-state EEG features and psychological traits (41). These findings provide preliminary multilevel associational evidence for the relationship between physical exercise and mental health and offer testable hypotheses for future causal research. However, given the multiple limitations in measurement methodology, study design, and data interpretation, all conclusions should be regarded as exploratory and preliminary, requiring validation through more rigorous experimental designs, more precise neural measurement technologies, and longitudinal tracking.

Therefore, although the bootstrap confidence intervals for the observed serial indirect effects did not include zero, these estimates may be unstable and should be interpreted cautiously. The present findings should be regarded as preliminary evidence of serial indirect associations rather than definitive evidence of multi-step mediating pathways. Future studies with larger, adequately powered samples are needed to replicate these associations. Moreover, the EEG-related serial indirect effects should be interpreted cautiously given their exploratory nature, limited statistical power, and reduced statistical support in covariate-adjusted sensitivity analyses.

## 5 Conclusion

There is a close correlation among physical activities, EEG-specific indicators and the state of flow. However, whether the EEG-specific indicators play a mediating role remains to be further verified.

## Data Availability

The raw data supporting the conclusions of this article will be made available by the authors, without undue reservation.
