# Supplementary material for: The association between depressive symptoms, physical activity, flow state, and resting-state EEG in college students
Source: Front Psychiatry. 2026 Oct 30;17:1903553. doi: 10.3389/fpsyt.2026.1903553 (PMC13759498; doi:10.3389/fpsyt.2026.1903553)
Supplement: Supplementary file 1 [file Supplementaryfile1.docx]

**SUPPLEMENTAL MATERIALS**

| **Appendix** | **Page** |
| --- | --- |
| Appendix-1 (Recruitment Flowchart) | 2 |
| Appendix-2 (Test Flowchart) | 3 |
| Appendix-3 (Correlation Coefficients between Depressive Symptoms, Flow State, Physical Exercise and Lateralization of Each Frequency Band in the Frontal Region) | 4 |
| Appendix-4 (Total effect and direct effect between physical exercise and depressive symptoms (via frontal pole δ lateralization and flow state)) | 5 |
| Appendix-5 (Specific Indirect Effects of Physical Exercise on Depressive Symptoms (via Prefrontal Delta Lateralization and Flow State)) | 6 |
| Appendix-6 (Total Effect and Direct Effect between Physical Exercise and Depressive Symptoms (via Frontal Pole Alpha 2 Lateralization and Flow State)) | 7 |
| Appendix-7 (Specific Indirect Effects between Physical Exercise and Depressive Symptoms (Through Frontal Pole Alpha 2 Lateralization and Flow State)) | 8 |

**Recruitment Flowchart**

**Test flowchart**

**Correlation Coefficients between Depressive Symptoms, Flow State, Physical Exercise and Lateralization of Each Frequency Band in the Frontal Region**

| **Variable** | **BDI-II** | FSS | **PARS-3** |
| --- | --- | --- | --- |
| BDI-II | 1 |  |  |
| FSS | -0.597^***^ | 1 |  |
| PARS-3 | -0.347^***^ | -0.514^***^ | 1 |
| Fp2/Fp1-δ | -0.354^***^ | 0.233^**^ | 0.176^*^ |
| Fp2/Fp1-θ | -0.374^***^ | 0.219^**^ | 0.133 |
| Fp2/Fp1-α1 | -0.390^***^ | 0.267^***^ | 0.132 |
| Fp2/Fp1-α2 | -0.428^**^ | 0.330^*^ | 0.143^*^ |

Note: ***p＜0.001, **p＜0.0, *p＜0.05

**Total effect and direct effect between physical exercise and depressive symptoms (via frontal pole δ lateralization and flow state)**

| **Effect size** | **Effect** | **SE** | **t** | **p** | **LLCI** | **ULCI** | **completely standardized effects** |
| --- | --- | --- | --- | --- | --- | --- | --- |
| Total effect | -0.3171 | 0.0586 | -5.4148 | <0.001 | -0.4325 | -0.2017 | -0.3471 |
| Direct effect | -0.1241 | 0.0512 | -2.4238 | 0.0162 | -0.2250 | -0.0232 | -0.1358 |

**Specific Indirect Effects of Physical Exercise on Depressive Symptoms (via Prefrontal Delta Lateralization and Flow State)**

| **Path** | **Effect** | **Boot SE** | **Boot LLCI** | **Boot ULCI** | **completely standardized effects** |
| --- | --- | --- | --- | --- | --- |
| TOTAL | -0.1930 | 0.0358 | -0.2687 | -0.1274 | -0.2113 |
| Ind1: Physical exercise → Fp2/Fp1-δ → Depression symptoms | -0.0342 | 0.0161 | -0.0697 | -0.0065 | -0.0375 |
| Ind2: Physical exercise → Flow state → Depression symptoms | -0.1446 | 0.0292 | -0.2083 | -0.0926 | -0.1582 |
| Ind3: Physical exercise → Fp2/Fp1-δ → Flow state → Depression symptoms | -0.0142 | 0.0070 | -0.0297 | -0.0023 | -0.0156 |

**Total Effect and Direct Effect between Physical Exercise and Depressive Symptoms (via Frontal Pole Alpha 2 Lateralization and Flow State)**

| **Effect size** | **Effect** | **SE** | **t** | **p** | **LLCI** | **ULCI** | **completely standardized effects** |
| --- | --- | --- | --- | --- | --- | --- | --- |
| Total effect | -0.3171 | 0.0586 | -5.4148 | <0.001 | -0.4325 | -0.2017 | -0.3471 |
| Direct effect | -0.1375 | 0.0503 | -2.7338 | .0068 | -0.2366 | -0.0383 | -0.1505 |

**Specific Indirect Effects between Physical Exercise and Depressive Symptoms (Through Frontal Pole Alpha 2 Lateralization and Flow State)**

| **Path** | **Effect** | **Boot SE** | **Boot LLCI** | **Boot ULCI** | **completely standardized effects** |
| --- | --- | --- | --- | --- | --- |
| TOTAL | -0.1797 | 0.0365 | -0.2574 | -0.1120 | -0.1967 |
| Ind1: Physical exercise → Fp2/Fp1-α2 → Depression symptoms | -0.0334 | 0.0177 | -0.0722 | -0.0011 | -0.0375 |
| Ind2: Physical exercise → Flow state → Depression symptoms | -0.1290 | 0.0263 | -0.1846 | -0.0817 | -0.1412 |
| Ind3: Physical exercise → Fp2/Fp1-α2 → Flow state → Depression symptoms | -0.0172 | 0.0087 | -0.0358 | -0.0006 | -0.0183 |
